# Knowing What We Know – Reflections on the Development of Technical Guidance for Loss Data for the Sendai Framework for Disaster Risk Reduction

**DOI:** 10.1371/currents.dis.537bd80d1037a2ffde67d66c604d2a78

**Published:** 2018-08-02

**Authors:** Lorcan Clarke, Kevin Blanchard, Rishma Maini, Alin Radu, Nuha Eltinay, Zehra Zaidi, Virginia Murray

**Affiliations:** Research Analyst InternPublic Health England; Public Health England; Public Health England; University of BristolUniversity of Bristol; School of Built Environment and Architecture, London South Bank University, London, United Kingdom; Institute for Risk and Disaster Reduction, University College London, London, United Kingdom; Public Health England, London, England; UNISDR Scientific and Technical Advisory Group, Geneva, Switzerland; Integrated Research on Disaster Risk Scientific Committee, Beijing, China

## Abstract

Introduction: To report on activities aligned with the Sendai Framework for Disaster Risk Reduction 2015-2030, national governments will use the Sendai Monitor platform to track progress using a series of indicators that inform seven Global Targets originally agreed in 2015. In February 2017, the UN General Assembly adopted a set of 38 agreed indicators based on work led by an open-ended intergovernmental expert working group (OIEWG) on indicators and terminology relating to disaster risk reduction. In January 2018 the United Nations Office for Disaster Risk Reduction released technical guidance documents in advance of the launch of the Sendai Monitor in March 2018. Methods: This paper discusses several challenges to recording and reporting on loss data under the Sendai Framework. Additional insights to elaborate on discussion build upon commentary and examples raised during a workshop held on developing loss data that was hosted by the United Nations Office of Disaster Risk Reduction (UNISDR), the Integrated Research on Disaster Risk (IRDR) programme, and Public Health England (PHE) from February 15-17 2017 at the Royal Society in London, United Kingdom. The meeting’s purpose was to refine technical guidance notes concerning Global Targets A, B, C, and D, which had been drafted in coordination with the work of the OIEWG. The workshop was attended by representatives from UN Agencies, UN Member States, international scientific bodies, academic bodies, the government of the United Kingdom and the private sector. Results: Global Targets A, B, C and D of the Sendai Framework have common and specific complexities which require acknowledgement and support in recording, reporting and using disaster loss data. Discussions during the February 2017 loss data workshop highlighted a number of complexities and the need for common standards and principles for loss data. Individual target complexities include attribution of health impacts, assessing impacts, consistently calculating economic losses and measuring disruption to critical infrastructure. Discussion: Transparent monitoring is critical to ensure political will, financial efforts and effective evidence support the global shift towards more sustainable development. Data involves common challenges which can undermine accuracy and understanding of reporting across the frameworks that outline the United Nations’ 2030 Agenda. Disaster loss data adds further challenges which require support and innovation to ensure stakeholders across sectors in all sectors have appropriate technical guidance that can support useful loss data management processes. The February 2017 workshop highlighted systemic challenges with working with loss data and highlighted several pertinent pathways to progress on the breadth and reliability of disaster loss data across different settings.

## 1. Introduction

The Sendai Framework for Disaster Risk Reduction 2015-2030 offers national governments an opportunity – to enhance their capacities to deal with disaster risk at all scales and across all sectors. It encompasses all hazards and disaster scenarios, including: small and large scale; frequent and infrequent; sudden- and slow-onset; caused by natural or man-made hazards; as well as related environmental, technological and biological hazards and risks.^1^ Along with complementary instruments of the United Nations’ 2030 agenda, such as the Sustainable Development Goals (SDGs) and the Paris Climate Agreement, the Sendai Framework offers UN Member States measures of progress.^2^ Seven agreed Global Targets focus on reducing: mortality, persons affected, economic loss, damage to critical infrastructure and disruption of basic services due to disasters; and improving local and national strategic disaster plans, international cooperation, and multi-hazard early warning systems and disaster-risk information and assessments.^1^

The development of the indicators against which to measure the Sendai Framework targets took place between September 2015 and November 2016. Development and refinement was led and undertaken by an open-ended intergovernmental expert working group (OIEWG) on indicators and terminology relating to disaster risk reduction. Formal meetings were held between 28 – 30 September 2015, 10 – 11 February 2016, and 15 – 18 November 2016. On 2 February 2017, the UN General Assembly adopted the OIEWG’s consensus on 38 indicators for use across the seven Global Targets.^3^^,^^4^ As of March 1 2018, the infrastructure for Member State reporting – the “Sendai Framework Monitor” – will provide the platform for measuring progress as part of the Sendai Framework Monitoring Process.^5^

There are significant challenges in the collection, recording and reporting of data. The Sendai Framework Readiness Review 2017 compiled the monitoring capabilities of 87 UN Member States, revealing significant heterogeneity between, and within, countries in their capacity to report against the approved indicators.^6^ This paper explores the utility of disaster loss data and examines the processes necessary to ensure monitoring under the Sendai Framework. Discussion in this paper also builds upon examples and commentary raised during a workshop on disaster loss data hosted by the United Nations Office for Disaster Risk Reduction (UNISDR), the Integrated Research on Disaster Risk (IRDR) programme, and Public Health England (PHE) between February 15 – 17 2017 at the Royal Society in London, United Kingdom.^7^ The workshop was attended by 44 participants in total, with representatives from UN Agencies, UN Member States, international scientific bodies, academic bodies, the government of the United Kingdom and the private sector (see Appendix 1).^7^

## 2. A place for indicators

The Millennium Development Goals (MDGs) Data Catalogue exemplified what was possible to better understand global problems and progress. Across the eight MDGs, data became available for directing funding and supporting political pressure that fed into local, regional and global progress outlined in the UN’s 2015 Millennium Development Goals Report.^8^^,^^9^ The need to support and account for progress catalysed better recording, reporting, and reviewing of information. Reliable data and evidence is crucial to effective policy making.^10^ By 2015, with the phasing out of the MDGs and the close of the Millennium Development Era, two challenges were apparent. There was invisibility and inequality in data across the 48 indicators that informed the eight MDGs. Datasets were incomplete geographically, temporally, and in terms of socio-demographic disaggregation.^11^

Building on the success and limitations of the Millennium Development Era, the United Nations 2030 agenda includes ambitious aims for data and monitoring. The alone include 18 goals, reported upon by more than 230 indicators, set for countries to achieve by 2030.^12^ The Sendai Framework for Disaster Risk Reduction 2015-2030 will map progress through seven Global Targets and 38 indicators.^3^ Ambition extends throughout the 2030 agenda via a call to “leave no one behind”. This phrase recognises the rights and dignity of all individuals in all countries and the need to target support towards those deemed “furthest behind” due to marginalisation or neglect of efforts.^13^ To understand who is “left behind”, it is critical to record and report disaggregated data. This requires robust information systems, accepted standards and technical guidance. However numerous countries cannot take reliably and specifically informed actions based on disaster loss data, due to unobtainable or aggregated information.^6^ Critical elements of domestic capacity include ability to collect, record, and report information at all levels. Acknowledging the fiscal and other limitations that exist, coordinating bodies such as UNISDR, can lead on two efficiency-promoting actions:

(1) Ensure complementarity between indicators across global instruments.

(2) Develop clear and contextually relevant technical guidance for data collection.^11^

Carrying out the former – ensuring complementary – has been apparent in the efforts of independent organisations and the independent expert working groups tasked with indicator development for the Sendai Framework.^4^^,^^14^^,^^15^ Ensuring in data efforts is central to the Sendai Framework Monitor, an online reporting mechanism, and coordinating progress assessments for the SDGs and the Sendai Framework.^16^ For instance, common goals are clear between several Sendai Framework Global Targets and SDG Goal 11 - “Make cities and human settlements inclusive, safe, resilient and sustainable”. Targets A and B require reporting on mortality, missing individuals, and affected persons for disasters; Targets C and D, which address damage and disruption to the build environment and critical infrastructure. All of these elements are critical to ensuring urban resilience, while Target E supports assessments of national and local disaster-risk strategies critical to urban resilience.^7^^,^^12^

Without accurate reporting, accepted and comprehensive indicators lose value. If the methods for recording and reporting on data are unclear, or perceived to give an inaccurate picture of reality, then opportunity for learning and progress may be lost.^17^ The Inter-agency and Expert Group (IAEG) on SDG Indicators and the OIEWG discussed and drafted recommendations of technical guidance which? informed the indicator refinement process, but these were? not included at the stage of acceptance for individual indicators.^3^^,^^18^ In January 2018 UNISDR released a collection of technical guidance notes for data and methodology to support the first cycle of monitoring using the Sendai Framework Monitor.^19^ However, further refinement is possible, particularly with respect to ensuring available capacity is appropriately harnessed and supported. The following analysis examines crucial issues present in assessing progress on Sendai Framework Targets A, B, C and D, using commentary and examples from the loss data workshop to elaborate.

## 3. Evaluating the state of loss data

Insights into disaster loss data in the context of the Sendai Framework are available in the aforementioned OIEWG Report, sector-specific examinations, and examinations of coherence with other instruments of the 2030 agenda.^3^^,^^4^^,^^14^^,^^20^ Analysis in this paper focuses on addressing comments raised during the loss data workshop on gaps in technical guidance and areas for further work to ensure countries can report against Sendai Framework Global Targets A, B, C and D.

**Table 1: Sendai Framework Targets A-D:** Targets A, B, C and D for the Sendai Framework for Disaster Risk Reduction 2015-2030.

Target	Description
Target A	Substantially reduce global disaster mortality by 2030, aiming to lower average per 100,000 global mortality rate in the decade 2020-2030 compared to the period 2005-2015.
Target B	Substantially reduce the number of affected people globally by 2030, aiming to lower average global figure per 100,000 in the decade 2020 -2030 compared to the period 2005-2015.
Target C	Reduce direct disaster economic loss in relation to global gross domestic product (GDP) by 2030.
Target D	Substantially reduce disaster damage to critical infrastructure and disruption of basic services, among them health and educational facilities, including through developing their resilience by 2030.

The Sendai Framework calls for country self-monitoring to assess progress.^1^ This implies that internationally comparative methods are not required and disaster loss data recording can take place using existing databases. This can ensure country capacities for recording and reporting are not overstretched and there is a focus on progress at the country level. Assessing the impact of hazardous events requires collaboration across national governments. Ministries devoted to health, business and the environment are a sample of those who will be key to accurately the extent and impact of biological and technological hazards. In the case of international comparisons, differences in technical capacities mean that the dispersion of results and potential presumptions of greater-than-calculated loss will vary. Nevertheless, loss data workshop participants agreed on the significant value of common standards and principles for loss data, including those applying across the Global Targets and corresponding indicators.

Coherent data principles for local and national reporting structures ensure that foundations for reporting on loss data are similar. The Sustainable Development Solutions Network Thematic Research Network on Data and Statistics (SDSN TReNDS) offers nine core principles to improve data quality and set the foundations for new data partnerships.^21^ In “Counting on the World” (2017), the following principles are proposed to support useful and usable contributions to the measurement of sustainable development:^21^

1. Data quality and integrity: Ensuring clear standards support the entire process of data design, collection, analysis and dissemination.
2. Data disaggregation: Informing, with appropriate safeguards in place, that data is disaggregated across dimensions including as geography, wealth, disability, sex, gender and age.
3. Data timeliness: Using standards and technology to reduce time between initial design of data collection and publication of statistics.
4. Data transparency and openness: Making all data on public matters or funded publically, including that produced by the private sector, open by default (with exemptions for genuine security or privacy concerns).
5. Data usability and curation: Designing data architecture that is user-oriented and user-friendly.
6. Protection and privacy: Developing and enforcing clear frameworks to regulate access and use of data.
7. Data governance and independence: Strengthening and protecting data quality through national statistical offices that are functionally autonomous from other government agencies.
8. Data resources and capacity: Investing in human capital, physical assets and technology to support governmental, intermediary and independent data systems.
9. (Human) data rights: Protecting human rights at the core of any mechanisms or entities set up to mobilize the data revolution for sustainable development.

If implemented, the above principles would ensure that recording and reporting of loss data has a common direction and cause. However, particular elements of loss data present more unique challenges. The following sub-sections examine issues and pathways to progress for reporting on Sendai Framework Global Targets A, B, C and D.

Target A

Loss of life severely disrupts the households and communities and is particularly felt by highly vulnerable, low-income groups in the context of disasters.^22^ Yet measuring mortality is challenging. The World Health Organization (WHO) regularly receives cause-of-death statistics from about 100 Member States, yet two-thirds (38 million) of 56 million annual deaths are still not registered.^23^ Workshop participants noted that the disruption associated with disasters adds to the challenge of registering mortality. Furthermore, Target A is also informed by an indicator of missing persons. Yet across different settings there is limited comparability and coordination on this matter. For example, in the United Kingdom, a person cannot be registered as missing and declared dead until 7 years afterwards, whereas in Italy, it is at least 10 years.^24^^,^^25^

Comprehensive attribution of mortality to disasters is complex. Alongside direct trauma or ill-health from infectious disease during health emergencies, there are many indirect impact pathways.^26^ Several examples were raised during the loss data workshop, including how during slow-onset hazards, such as droughts, health effects may be mediated through the disruption to basic healthcare services and spread of communicable diseases.^27^ Participants agreed that technical guidance and work to improve loss data capacity should harness the available evidence to identify common and applicable causes of death from different types of hazards. This also raises the question of how to best classify hazards, as different taxonomies exist across different settings and there is no commonly accepted standard. Furthermore, time periods between the exposure to hazards and death can vary widely. Disruption of care for chronic conditions and onset of persistent stress can lead to greater disease burden or even death that may not occur for months or years after a disaster.^28^

Engaging broader systems for assessing mortality may offer another avenue to support disaster loss data management for health. The Global Burden of Disease (GBD) study, led by the Institute for Health Metrics and Evaluation, offers a platform to better assess disaster-related mortality using advanced modelling approaches.^29^ The GBD study is the most comprehensive worldwide epidemiological study in existence, with a description of mortality from a variety of causes at global, national and regional levels. The extraction of baseline health measurements for some of the SDGs from the GBD is already being explored.^30^ In addition, the World Health Organization’s ‘Global Reference List of 100 Core Health Indicators’ collates comprehensive reported information and aims to contribute to greater alignment between countries on the reporting of health trends.^31^ Whereas initiatives such as “The Lancet Countdown: Tracking Progress on Health and Climate Change” provide insights about an array on influential outcomes from and causes of disruption to health and health care.^32^

Target B

Each year between 2006 and 2016 an estimated 224 million people were affected by disasters attributed to natural hazards alone.^33^ Better understanding of impacts upon livelihoods is critical to reducing welfare impacts, especially in light of World Bank estimates that losses from shocks to economic activity from disasters amounts to US$520billion.^22^ Along with the acceptance of the indicators for the Sendai Framework Global Targets, United Nations Resolution 71/276 also accepted the definition affected people to include individuals that have sustained injuries or illness, whose houses have been damaged or destroyed, or those who have experienced disruption to their livelihoods as a result of a disaster event.^3^ As with Target A, concerns around attribution apply. Target B encompasses scenarios where cascading effects from hazards can develop into significant impacts. A simple assessment approach is critical, as measurement involves drawing information from a wide range of sectors.

Discussions at the February 2017 loss data workshop focused on establishing examples of harnessing existing systems of measurement for persons affected. Similar to Target A, data on injured and ill people can come from existing health indicators that are adapted to target disaster specific impacts. However, clear clarification is essential for periods of time use for measurement and the inclusion of secondary illness and injury. Mental health issues, amongst the most acute health impacts associated with disasters, are a specific area requiring definition within ill and injured person calculations. Geographic information systems (GIS) and remote sensing techniques can assess impacts to the physical environment, such as dwellings and local infrastructure, however local authorities and international standards needs to also account for degrees of damage to informal settlements. Further discourse noted the value of establishing proxies for assessing impacts to affected persons. Such methods are used by actors including the World Bank Groups’s Global Facility for Disaster Reduction and Recovery (GFDRR), which has employed post-disaster needs-assessment techniques using sector-specific data for employment, agriculture, health, transport, and communication to calculate the impact of disasters on human well-being.^34^ Moreover, the UN Food and Agricultural Organization (FAO) has previously estimated the livelihood impact of disasters using data on agriculture, food security and nutrition.^35^

Target C

In agreed indicators, “economic loss” encompasses value in the following categories: agricultural, productive, housing, critical infrastructure, and cultural heritage. The term “direct”, based on guidance of the OIEWG, refers to losses in assets. Despite progress during the implementation of the Hyogo Framework in building physical resilience to disasters, economic losses remain substantive. The GFDRR estimates that global annual losses attributed to disasters amount to over $300bn in asset stock. 22 This definition omits the substantial losses in productivity and well-being which lead to economic impact, however the complexity of necessary assessment protocols was avoided to ensure that indicator calculation was practical and feasible.^3^ Measurements for assessment of indirect economic losses are less developed and not included in the Sendai Framework. But understanding the cascading impacts of disasters on economic welfare and productivity is critical, particular as drivers of hazard risks changes over time.^3^^,^^26^

Economic loss assessments by member states will engage a broad cross-section of actors. These include international institutions (e.g. the World Bank and UNISDR), private sector companies expertise (e.g. insurance and catastrophe risk modelling industries) and national governmental bodies.^19^^,^^22^ Loss data workshop participants noted collaboration in this area can build on and support existing cooperation between public actors and risk transfer supply chains composed of catastrophe-modelling firms, primary insurance companies, and reinsurance providers.^36^ The World Bank’s Disaster Risk Financing and Insurance Program (DRFIP) is one example of a public-private partnership. DRFIP aims to reduce economic disruption by supporting prompt government responses to disasters that does not compromise sovereign fiscal balances.^37^

Reliable and consistent economic-loss calculations practices are critical for disaster loss data. At the loss data workshop, discussions highlighted the value of improving transparency in methods between private actors (e.g. catastrophe modelling companies) and accounting for geographical and temporal price fluctuations. When reliable information is absent proxies may be useful, but come with the caveat that non-private price indices are used as often as possible; an example of this is reconstruction inputs such as building materials. Noted challenges extend to the application of affected ratios (i.e. amount of damage due to a hazard) that may give binary, categorised (segmented), or continuous (percentage) values in damage ratios. At different periods following a hazard impact, reporting practices should also reflect need. Such rapid assessment protocols for soon after hazard impact, and another, more accurate, record of direct economic loss several c.1 year after a hazardous event. Estimating losses to cultural heritage, which inform indicator C6, are a unique and context specific challenge. While available guidance proposes assignment for non-movable and movable cultural heritage assets, their value is difficult to disentangle from local connection and (if applicable) tourism related income.^19^ Stakeholders also made it clear that cultural heritage issues associated with the natural environment further adds to this challenge.

Target D

Critical infrastructure comprises ‘physical structures, facilities, networks and other assets which provide services that are essential to the social and economic functioning of a community or society’.^3^ These factors play a critical role in how communities and systems will cope with hazardous events. If severe disruption takes place, emergency capacities are critical to mitigate disruption to essential services such as health care, education and transportation. Maintaining societal functions and productive capacities offsets financial and welfare risks in the short and medium term due to damage and disruption attributed to hazardous events. Strengthening key facilities is a key principle of locally led and internationally coordinated programs such as the Comprehensive Safe Hospitals Framework and the Worldwide Initiative on Safe Schools.^38^^,^^39^

Disruption to basic services may not require damage or destruction to infrastructure. Technological hazards include those which disrupt information systems, such as threats to cybersecurity. Across countries, computers are critical to continuity across basic service sectors. In March 2017, the global spread of “WannaCry” ransomware revealed the vulnerability of the UK’s National Health Service, causing disruption to clinical care and trust in the security of health record systems.^40^ The June 2017 “Petya” malware spread in Ukraine infected several elements of state infrastructure including energy, finance and government ministries.^41^

Target D has similarities with Target C that echo those between Target A and Target B. UNISDR technical guidance for monitoring and reporting recommends calculating indicators D-1 to D-4 using the same data and critical infrastructure units and facilities as C-3 and C-5. Common metadata formats are also recommended across C-5/D-4 and C-3/D-8.^19^ Furthermore, clear definitions are key to consistency in reporting on Target D. For instance, loss data workshop participants noted the challenges of measuring disruption due to slow-onset and small-scale disasters. Contrary to recommendations, damage and disruption to infrastructural assets and services can be disaggregated according to the institutional level e.g. primary or secondary health facilities, rather than based upon size. Such classifications are in line with practices in public sector risk assessment and private sector catastrophe modelling used to inform insurance products.^42^^,^^43^^,^^44^

## 4. Conclusion

Transparent monitoring is essential to ensure that political and financial efforts to implement the 2030 agenda align with accepted goals and foster an effective evidence base. It is critical to offer support to the institutions tasked with doing so. Technical guidance literature is an essential part of this, which the February 2017 loss data workshop and recent publication of UNISDR technical guidance have revealed. Across Sendai Framework Global Targets A-D, there remain specific issues that the academic community can support with innovating methods for improving estimation and accurate record of the impacts of disasters. The February 2017 disaster loss data workshop provided a pointed moment to reflect and exemplify these issues among a diverse range of stakeholders, from different regions and sectors.

UN member states have accepted the Sendai Framework Global Targets and their component indicators. To move forwards on implementation and monitoring of progress, country statistical offices and stakeholders need support to develop reliable loss data recording, reporting and analysis capacities that provide useful and usable information. However, disaster loss data has problematic characteristics common to all data. With further specific reliability and coverage challenges that, if not acknowledged and addressed, can undermine comprehensive understanding of what has happened, what could happen and what to do about it.

## Competing Interests Statement

The authors have declared that no competing interests exist. Prof. Virginia Murray serves on the Editorial Board of PLOS Currents: Disasters.

## Data Availability Statement

All relevant data are within the paper. No quantitative data was recorded for the purposes of this paper. Workshop discussions and findings were synthesised from rapporteur reports delivered during and after the workshop. Attendee affiliations outlined in Appendix 1.

## Corresponding Author

Lorcan Clarke - clarkel5@tcd.ie

## Appendix 1: Outline of Workshop on Disaster Loss Data – Held 15-17 February 2017 in London, UK.

In light of the value of developing the technical guidance for indicators and to build on previous efforts, UNISDR, IRDR, and PHE coordinated a three-day Loss Data Workshop in London, United Kingdom from 15 to 17 February 2017. The aim of the workshop was to advance and support production of technical guidance loss data informing Sendai Framework Global Targets A, B, C, and D.^7^ This guidance seeks to inform how UN Member States can collect, record and report under the Sendai Framework for Disaster Risk Reduction. The workshop occurred following after ratification by the UN General Assembly of the OIEWG recommended indicators to report on Sendai Framework’s Global Targets on 2 February 2017, and during the lead-in period to the 5th Global Platform for Disaster Risk Reduction in May 2017.^4^ The workshop was attended by 44 participants in total, with representatives from UN Agencies, UN Member States, international scientific bodies, academic bodies, the government of the United Kingdom and the private sector. For this consultation, workshop organisers invited stakeholders across public, private and multilateral agencies. Discussions took place under the Chatham House rule. This ensured anonymity was given to participants’ contributions and provided an open environment for pragmatic discussion. 45 Participants were split across several working groups that had assigned rapporteurs to report back to all attendees from breakout sessions.

Organizations represented at the loss data workshop included the following (sorted by type): UN Member States: Japan, Fiji, Ecuador, Indonesia, Zambia, Zimbabwe. International Scientific Bodies: Joint Research Centre of the European Commission, Pacific Community, Committee on Data for International Council for Science. Government of the United Kingdom: Cabinet Office, Public Health England, Office for National Statistics, Department for International Development, Environment Agency. Academic Sector: UK Research Council, King’s College London, University of Bristol, University College London, London South Bank University.

Rapporteurs were selected by organisers from multi-disciplinary backgrounds and varying levels of experience in academia, industry, or non-profit organisations. These diverse perspectives benefited proceedings through rapporteurs’ ability to actively contribute to and provide context for discussion notes among the working groups assigned to targets. For instance, those with a background in public health worked on Target A, while those with experience in economics or engineering meant were assigned to Targets C and D. Beyond this, rapporteurs were tasked with the preparation of workshop documents, integrating comments and recommendations into revised technical-guidance notes for the Global Targets under review. Additionally, the rapporteurs produced an overarching “essential reading” document to promote clarity for attendees and users of the technical guidance. These documents were then further developed by UNISDR in advance of the Global Platform for Disaster Risk Reduction in May 2017. Where further efforts were made to develop “essential reading” documents.

During the workshop preparation process it emerged that the role of rapporteurs is not set out in accepted and accessible guidance documents. The role of a rapporteur is context specific and can encompass various meanings. At international institutions, such as the European Parliament, rapporteurs are appointed to lead investigations and report back to the assigning body.^46^ The United Nations “Special Rapporteurs” take on a similar role, for example via appointment by the Office of the High Commissioner for Human Rights to investigate specific relevant issues. 47 However openly published guidance for rapporteurs appointed to assist in a meeting or workshop is not available. This is concerning due to the ubiquity of the role and its utility within multilateral systems of governance. The resulting lack of clarity, for how meeting reports are produced and published, then inhibits understanding of how openly available documents of international institutions come to exist. Although this paper does not attempt to define the role of a rapporteur in this context, it highlights a need for guidance to ensure defined good practice is applied in future events.
