# Correction: Knowing What We Know – Reflections on the Development of Technical Guidance for Loss Data for the Sendai Framework for Disaster Risk Reduction

**DOI:** 10.1371/currents.dis.e597ed667989b083254fefcac8853875

**Published:** 2018-09-24

**Authors:** - PLOS Currents

## Correction

The affiliation for the sixth author is incorrect. Zehra Zaidi is affiliated with the Euro-Mediterranean Center on Climate Change and Ca' Foscari University of Venice.

The following information is missing from the Funding section: ZZ was supported by the SMALLDIS project, which received funding from the European Union's Horizon 2020 research and innovation programme under the Marie Sklodowska-Curie grant agreement No. 707683.
